# Regulation of autophagy by protein lipidation

**DOI:** 10.1007/s44307-024-00040-w

**Published:** 2024-09-20

**Authors:** Yuqian Shao, Junchao Hu, Huihui Li, Kefeng Lu

**Affiliations:** 1 Department of Neurosurgery, State Key Laboratory of Biotherapy, West China Hospital, Sichuan University, Chengdu, 610041 China; 2 Department of Pathology, West China Second University Hospital, Sichuan University, Chengdu, 610041 China

**Keywords:** Autophagy, Lipidation, S-palmitoylation, N-myristoylation, S-prenylation, Glycosylphosphatidylinositol (GPI) anchor, Cholesterylation

## Abstract

Autophagy is a conserved catabolic recycling pathway that can eliminate cytosolic materials to maintain homeostasis and organelle functions. Many studies over the past few decades have demonstrated that abnormal autophagy is associated with a variety of diseases. Protein lipidation plays an important role in the regulation of autophagy by affecting protein trafficking, localization, stability, interactions and signal transduction. Here, we review recent advances in the understanding of the role of lipidation in autophagy, including S-palmitoylation, N-myristoylation, S-prenylation, glycosylphosphatidylinositol (GPI) anchor modification and cholesterylation. We comprehensively review the enzymes and catalytic mechanisms of lipidation and discuss the relationship between lipidation and autophagy, aiming to deepen the understanding of lipidation and promote the discovery of drug targets for the treatment of autophagy-related diseases.

## Introduction

Autophagy is a conserved catabolic recycling pathway that can eliminate cytosolic materials to maintain protein homeostasis and organelle functions. Protein lipidation, including S-palmitoylation, N-myristoylation, S-prenylation, glycosylphosphatidylinositol (GPI) anchoring and cholesterylation, plays an important role in the regulation of autophagy. Recently, numerous studies have demonstrated that lipidation can regulate the functions of ATG proteins and other key components of autophagy. The relationship between protein lipidation and autophagy has emerged as an exciting area of research.

Here, we review recent advances in the understanding of how protein lipidation regulates autophagy. We then primarily summarize the relationship between protein lipidation and autophagy, aiming to provide a comprehensive reference for research on the lipidation of autophagy-related proteins.

### Autophagy and protein lipidation

**Fig. 1:**
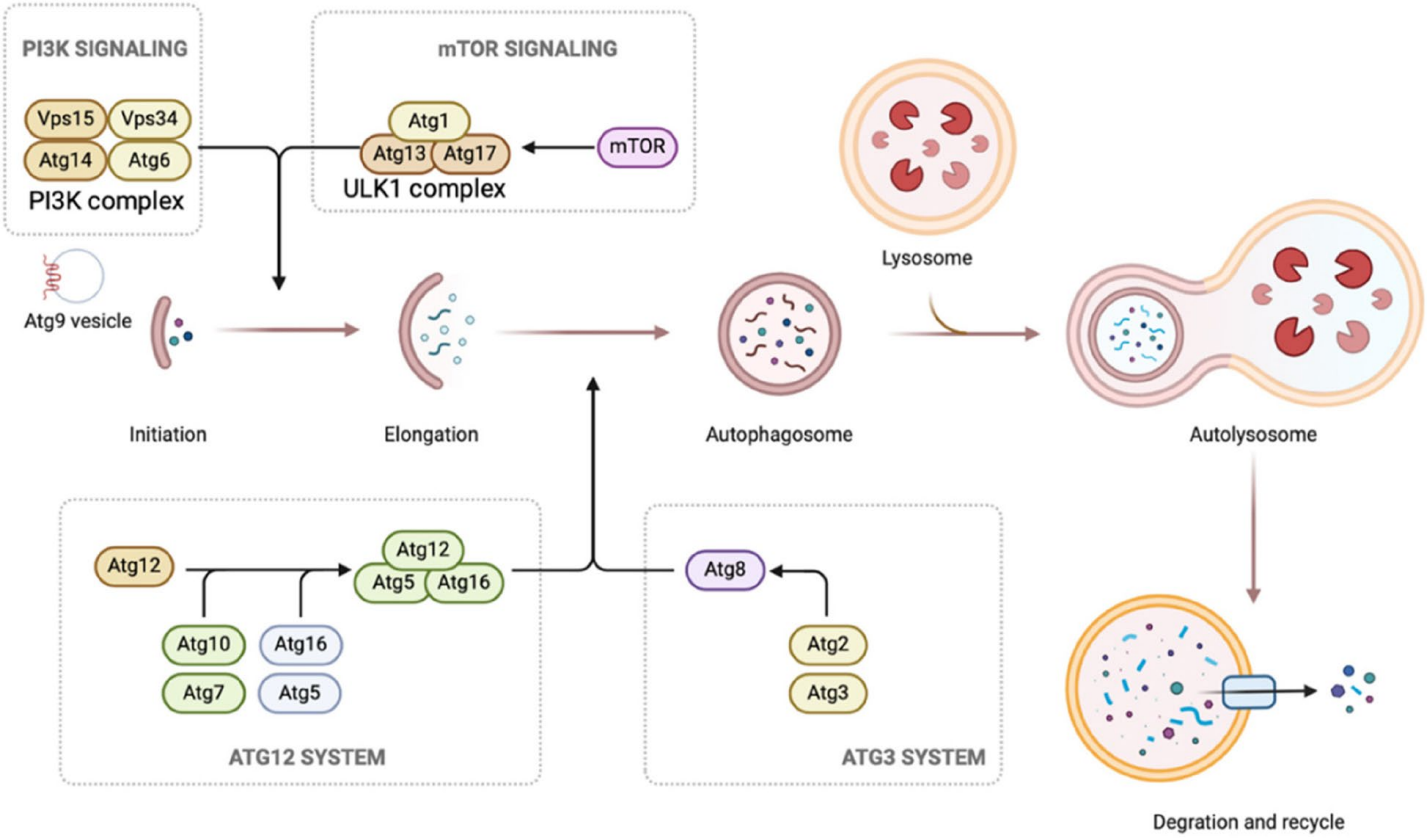
The process of autophagy. (1) Initially, phosphatidylinositol 3-phosphate (PtdIns3P) is responsible for the recruitment of downstream effectors. Simultaneously, ATG9 vesicles are generated. (2) The ULK complex is recruited to the autophagosome formation site and triggers nucleation of the separation membrane. (3) The Atg12 coupling system, which includes the E1 enzyme Atg7 and the E2 enzyme Atg10, catalyses the coupling of the ubiquitin-like protein Atg12 to Atg5. The Atg12–Atg5 conjugates further interact with Atg16. (4) The Atg3 coupling system is composed of Atg8 and Atg2. The Atg12 and Atg3 coupling systems function in multiple steps of autophagy, including the elongation and closure of the membrane, reorganization and selection of cargo, etc. (5) The contents of autolysosomes are degraded and recycled

Autophagy is an evolutionarily conserved catabolic process among eukaryotes that involves lysosomal degradation and can be divided into three main types: macroautophagy, microautophagy and chaperone-mediated autophagy (CMA)(Wang et al. 2022, 2023; Abdrakhmanov et al. 2020; Arias and Cuervo 2020; Chino and Mizushima 2020). Normally, autophagy refers to macroautophagy (Fig. 1), which involves a series of autophagy-related (ATG) proteins(Yamamoto et al. 2023) involved in the initiation of autophagy, membrane elongation, membrane closure, lysosomal fusion and degradation(Kuchitsu and Taguchi 2023). Many studies have demonstrated that autophagy dysfunction is associated with a variety of diseases such as cancer, neurodegenerative diseases, immune diseases, and ageing(Klionsky et al. 2021; Mizushima and Levine 2020; Debnath et al. 2023).

The function of autophagy is regulated by various protein modifications such as phosphorylation(Zhou et al. 2019; Udupa et al. 2023; Wu et al. 2023; Lee et al. 2023), glycosylation(Yang et al. 2023; Choi et al. 2023; Wei et al. 2023), ubiquitination(Shim et al. 2023; Lu et al. 2023), methylation(Ma et al. 2023; Jia et al. 2023), acetylation and protein lipidation (Zhong et al. 2020; Xie et al. 2015). Among these modifications, protein lipidations are among the most important protein modifications that regulate autophagy. These modifications include five main types: N-myristoylation, S-palmitoylation, S-prenylation, glycosylphosphatidylinositol (GPI) anchoring and cholesterylation(Yuan et al. 2024, 2020; Ko and Dixon 2018; Palsuledesai and Distefano 2015; Hu et al. 2022; Chen et al. 2018; Kallemeijn et al. 2021). These lipidations participate in the initiation, elongation, the formation of autophagosomes and the fusion with lysosomes.

## N-myristoylation

### N-myristoylation and N-myristoyltransferase

Recent studies have revealed that N-myristoylation plays a vital role in the regulation of autophagy by modulating protein trafficking, localization, stability, interactions and signal transduction(Kosciuk et al. 2020; Jia et al. 2023; Sun et al. 2022; Jacquier et al. 2018; Chen Y-C, Navarrete M S, Wang Y,, et al. 2020; Udenwobele et al. 2017).

**Fig. 2:**
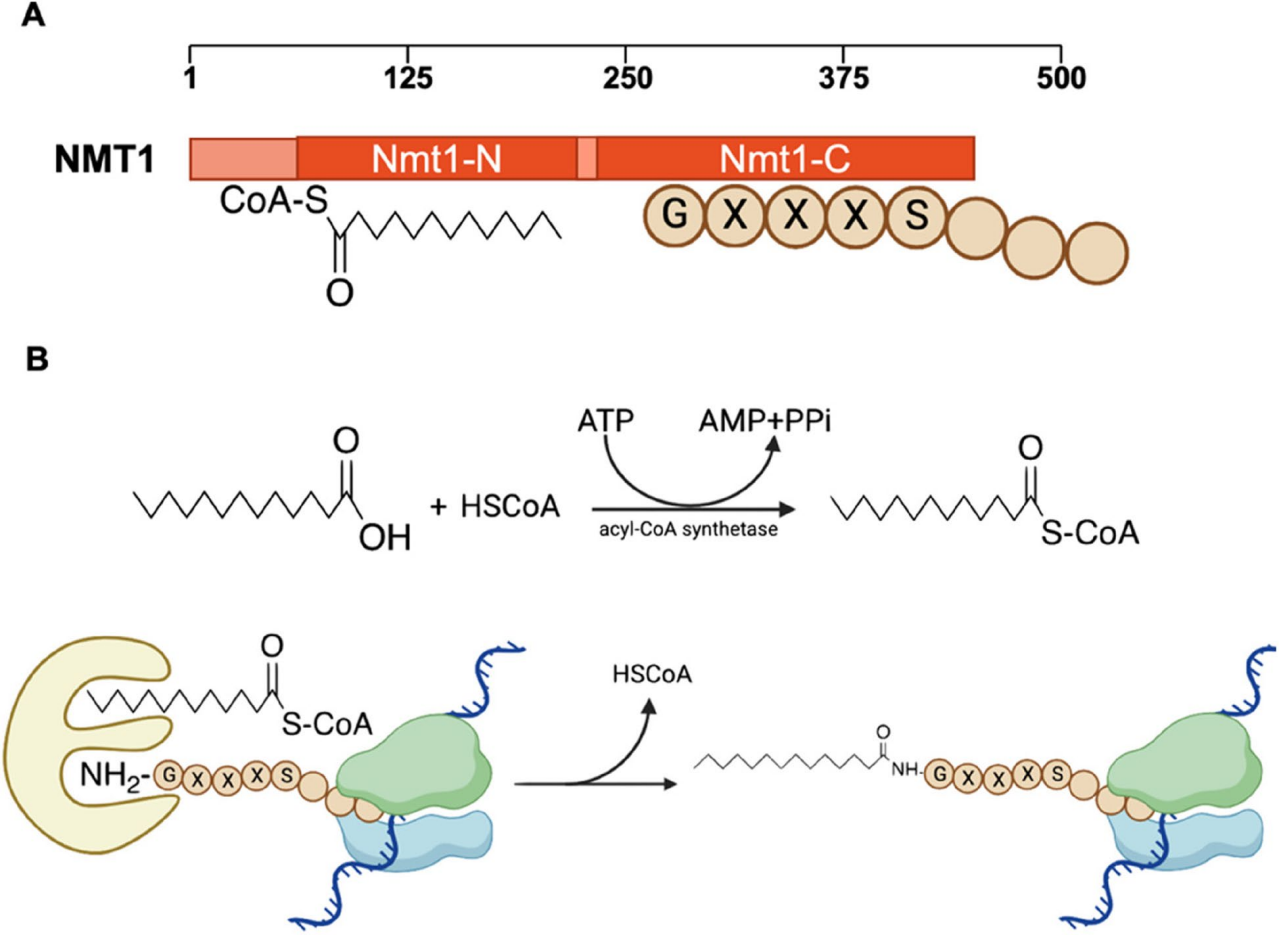
NMTs and N-myristoylation. (**A**) The structure and function of NMT1. (**B**) The process of N-myristoylation

N-myristoylation is a critical type of lipidation catalysed by N-myristoyltransferase (NMT)(Bhatnagar et al. 1998), which is a ubiquitous enzyme in eukaryotes. Lower eukaryotes, such as *S. cerevisiae*, contain only one isoform of NMT, whereas most mammals express two isozymes: NMT1 and NMT2(Thinon et al. 2014). Various studies on crystal structures have reported that NMT contains two main conserved domains(Castrec et al. 2018; Meinnel et al. 2020; Dian et al. 2020): NMT-N binds to myrisoyl-CoA binding pockets, whereas NMT-C can recognize and bind the consensus motifs of substrates (Fig. 2A). On the basis of the structures of the human NMT1 protein, which was crystallized with reactive substrates, and the sequences of N-myristoyl proteins, the consensus myristoylation motif is Gly-XXX-Ser (where X represents any amino acid)(Meinnel et al. 2020; Johnson et al. 1994). Several studies have shown that N-myristoylation (Fig. 2B) occurs via a Bi-Bi mechanism(Yuan et al. 2020; Johnson et al. 1994). First, myristoyl-CoA is formed under the catalysis of acyl-CoA synthetase. Next, myristoyl-CoA binds apo-NMT and exposes the substrate pocket. After the formation of an NMT-myristoyl-CoA-peptide complex, NMT can catalyse the formation of amide bonds between N-glycine and myristic acid. NMT subsequently releases the N-myristoyl protein and HSCoA, after which NMT recovers from conformational changes and conceals the substrate pocket.

In most cases, N-myristoylation occurs on glycine residues via an irreversible amide bond. However, in rare cases, myristic acid can attach to a lysine residue(Dian et al. 2020) via glycerol thioester and ester linkages(Yuan et al. 2024; Stevenson et al. 1992; Lin et al. 2012), which can be removed by human sirtuin 2 (SIRT2)(Teng et al. 2015). Moreover, the Shigella virulence factor Ipaj can reverse the N-myristoylation of proteins(Burnaevskiy et al. 2013).

### N-myristoylation of autophagy-related proteins

N-myristoylation(Towler et al. 1987), which is pivotal in the regulation of protein activity, was first reported in 1987. Several researchers have reported that NMT1 can influence the process of autophagy via the mTOR pathway(Sun et al. 2022; Chen Y-C, Navarrete M S, Wang Y,, et al. 2020), the STING pathway(Jia et al. 2023) and the JNK pathway(Deng et al. 2018). Typically, myristoylation influences the stability of proteins and protein-membrane interactions for proper protein localization and biological function. Here, we focused on the function of the N-myristoylation of autophagy-related proteins (Table 1).

**Table 1:** Proteins subjected to N-myristoylation that regulate autophagy

lipidation	autophagy-related protein	autophagy	disease
N-myristoylation	Huntingtin protein(Martin et al. 2014)	up	huntington disease
	AMPKβ(Liang, et al. 2015)	up	lung cancer
	LAMTOR1(Chen Y-C, Navarrete M S, Wang Y,, et al. 2020)	up	Lewis lung carcinoma
	LAMTOR1(Sun et al. 2022)	down	bladder cancer
	ARF1(Jia et al. 2023)	up	HSV-1 infection-induced innate antiviral immunity
	VPS15(Nemazanyy et al. 2013)	unknown	AVM

#### N-myristoylation of the Huntingtin protein is necessary for the formation of autophagic vesicles

Huntington disease (HD) is a degenerative neurological disorder that results in the aggregation of proteins. Owing to N-myristoylation, HTT_553-585_ can be correctly localized in the autophagosome. In contrast, blocking N-myristoylation of HTT_553-585_ impaired the formation of autophagic vesicles(Martin et al. 2014). This study provides new ideas for treating HD by modulating the N-myristoylation of HTT_553-585_.

#### N-myristoylation of LAMTOR1 regulates autophagy via the mTOR pathway

LAMTOR1 (late endosomal/lysosomal adaptor MAPK and mTOR activator 1) is an adaptor of the lysosome membrane(Malek et al. 2012). Chen et al. reported that the downregulation of NMT1 leads to decreased N-myristoylation of LAMTOR1, thereby suppressing the degradation of lysosomes and blocking the activation of mechanistic target of rapamycin complex 1 (mTORC1) to inhibit autophagy in Lewis lung carcinoma cells(Chen Y-C, Navarrete M S, Wang Y,, et al. 2020). However, Sun et al. reported that impaired N-myristoylation of LAMTOR1 affects the stability of LAMTOR1 by circumventing its ubiquitination-mediated degradation and altering its lysosomal localization, which is critical for the initiation of autophagy via the mTOR pathway in bladder cancer cells(Sun et al. 2022). The exact relationship between the N-myristoylation of LAMTOR1 and autophagy is worthy of in-depth study.

#### N-myristoylation of ARF1 promotes STING activation-triggered autophagy

Adenosine diphosphate-ribosylation factor 1 (ARF1) functions in intracellular protein transport to or within the Golgi apparatus(Stearns et al. 1990), and N-myristoylation of ARF1 was detected in 2013(Burnaevskiy et al. 2013). A recent study verified that myristic acid enhances the N-myristoylation of ARF1, a critical regulator of the membrane trafficking of STING, which combines with LC3 to form autophagosomes, facilitating STING activation-triggered autophagy to limit cGAS-STING-induced IFN responses (Jia et al. 2023). These findings suggest that N-myristoylation is a promising target for the treatment of herpes simplex virus-1 (HSV-1) infection-induced innate antiviral immunity.

#### N-myristoylation of AMPKβ is vital for mitophagy

In response to energy stress, the LKB1-AMPK cascade can mediate starvation-induced autophagy(Bakula et al. 2018). Xu provided several lines of evidence that AMPK is important for the selective removal of damaged mitochondria through autophagy(Liang, et al. 2015).They reported that N-myristoylation takes control of the initiation phase during selective autophagy-mediated membrane association(Liang, et al. 2015). In other words, N-myristoylation of AMPKβ is critical for the recruitment of AMPK to the mitochondria to induce mitophagy, which is highly important for maintaining cancer cell viability. Hence, it is a potential therapeutic target for cancer treatment.

#### N-myristoylation of Vps15 and the PI3K complex

The PI3K complex can recruit downstream factors for the initiation of autophagy, consisting of Vps34, Vps15, and beclin1. Among these, Vps15 is an essential component of the PI3K complex, which is critical for the recruitment of Vps34 to the membrane and subsequent stimulation of Vps34p PI3K activity(Stack et al. 1995, 1993). Moreover, the activity of the Vps34/Vps15 complex is critical in disease conditions, such as AVM and other lysosome storage diseases(Nemazanyy et al. 2013). Herman demonstrated that the Vps15 protein can be myristoylated at its N-terminus by labelling Vps15 with [^3^H] myristic acid(Herman et al. 1991). However, the exact relationship between N-myristoylation of Vps15 and autophagy remains elusive.

## S- palmitoylation

### S- palmitoylation and DHHC-PATs

Palmitoylation is catalysed by palmitoyltransferases called DHHC palmitoyl acytransferases (DHHC-PATs)(Mitchell et al. 2006), which share a conserved zinc-finger DHHC domain(Stix et al. 2020; Greaves and Chamberlain 2011). According to the different chemical bonds attached to proteins, palmitoylation can be divided into three types: S-palmitoylation linked by thioester bonds, N-palmitoylation linked by amide linkages, and O-palmitoylation linked by Oxy-ester bonds(Fan et al. 2024; Gao and Hannoush 2014; Nakao et al. 2002). Among these, S-palmitoylation is a typical reversible type of lipidation owing to the instability of the thioester bond(Won and CHEUNG SEE KIT M, MARTIN B R. 2018; Howie et al. 2014).

**Fig. 3:**
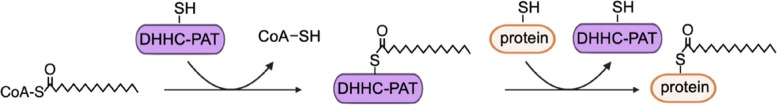
Process of S-palmitoylation

Previous studies have verified that S-palmitoylation also involves Ping-Pong mechanisms(Jennings and Linder 2012). First, DHHC-PATs undergo self-palmitoylation and the saturated C_16_ fatty acid is attached to DHHC-PATs, which are called acyl intermediates; then, the saturated C_16_ fatty acid is transferred to the substrate protein (Fig. 3). In response to upstream signals, this modification can be reversed by three main types of thioesterases, namely, APTs, palmitoyl-protein thioesterases (PPTs) and ABHD17 family thioesterases(Won and CHEUNG SEE KIT M, MARTIN B R. 2018; Azizi et al. 2019; Caswell et al. 2022).

### S-palmitoylation and autophagy-related proteins

Various studies have shown that the S-palmitoylation of proteins affects mainly protein-membrane associations(He et al. 2023; Sanders et al. 2019). The S-palmitoylation is vital in the progression of autophagy and many autophagy-related proteins have been identified over the last few years (Table 2).

**Table 2:** Proteins subject to palmitoylation that regulate autophagy

lipidation	autophagy-related protein	autophagy	disease
palmitoylation	ARMC3(Lei et al. 2021)	up	spermatogenesis
	NOD2(Zhou et al. 2022)	up	inflammatory diseases
	p62(Huang et al. 2023)	up	neurodegenerative diseases
	CD36(Li et al. 2023)	up	septic liver injury
	beclin1(Guo et al. 2024)	up	Alzheimer's disease
	mTOR1(Panwar et al. 2023)	unknown	ageing, neurological diseases, and human malignancies

#### Palmitoylation of nucleotide-binding oligomerization domain protein 2 (NOD2) increases the stability of NOD2 and modulates inflammation

NOD2 can sense bacterial peptidoglycan and induce proinflammatory and antimicrobial responses(Cooney et al. 2010). Zhou et al. reported that the palmitoylation of NOD2 impairs the association between NOD2 and the cargo receptor SQSTM1/p62, thereby suppressing the degradation of NOD2, which is pivotal for sensing bacterial peptidoglycan and inducing proinflammatory effects(Zhou et al. 2022).

#### Palmitoylation of ARMC3 facilitates PIK3C3-C1 recruitment and Ptdins3P generation to regulate autophagy

During the initiation of autophagy, class III phosphatidylinositol 3-kinase complex I (PtdIns3K-CI) is recruited to the membrane of the vacuole and generates phosphatidylinositol-3-phosphate (PtdIns3P), which is essential for the formation of autophagosomes. Lei et al. confirmed that the palmitoylation of ARMC3 at Cys507 and Cys518 facilitates PIK3C3-C1 recruitment and Ptdins3P generation to construct autophagosomes(Lei et al. 2021). This process provides energy for flagellar motility during spermiogenesis, which is essential for sperm motility and fertility(Lei et al. 2021).

#### Activation of mTOR is palmitoylation-dependent

Mammalian target of rapamycin (mTOR) is important for sensing a variety of extracellular signal stimuli and participates in autophagy and apoptosis, among other processes(Mordier et al. 2000). Sanders et al. reported that mTOR signalling is palmitoylation-dependent(Sanders et al. 2019). An increasing number of studies have revealed that constitutive activation of the mTOR pathway is responsible for ageing, neurological diseases, and human malignancies(Panwar et al. 2023). Nevertheless, few studies have described the mechanism by which palmitoylated mTOR affects the process of autophagy(Panwar et al. 2023).

#### Palmitoylation of beclin1 promotes the initiation of autophagy

The S-palmitoylation of the autophagic core protein beclin1, which is modified by DHHC5 at Cys137, initiates autophagy. Guo et al. reported that the palmitoylation of beclin1 mediated by DHH5 promotes hydrophobic interactions between beclin1 and the adaptor proteins ATG14L and VPS15, which specifically promote the formation and activity of the autophagic core complex PI3KC3-C1(Guo et al. 2024). However, the loss of DHHC5 in neurons leads to the accumulation of tau and Aβ aggregates in the PS19 and 5 × FAD mouse models of AD, respectively, in an autophagy-dependent manner(Guo et al. 2024).

#### Depalmitoylation of CD36 impacts the fusion of autophagosomes and lysosomes

Cluster differentiation-36 (CD36) is a pivotal protein that governs fatty acid metabolism, autophagy and immunity and is related to metabolic diseases, cardiovascular conditions, and cancer(Yang et al. 2024). Research has shown that the depalmitoylation of CD36 leads to the distribution of CD36 from the plasma membrane to lysosomes, thereby promoting the proteasomal degradation of SNARE proteins in a UBQLN1-dependent manner and resulting in fusion impairment; thus, the palmitoylation of CD36 is a promising therapy for treating septic liver injury(Li et al. 2023).

#### Palmitoylation of the receptor p62 promotes autophagy

Sequestosome 1 (SQSTM1)/p62 is an autophagy receptor that captures cargoes into autophagosomes. Moreover, the S-acylation of p62 at Cys289 and Cys290 promotes the formation of small p62 droplets, which enhances the autophagic membrane localization of p62 droplets, facilitating efficient degradation of their substrates(Huang et al. 2023).

S-palmitoylation is reversible when it is catalysed by the enzyme ZDHHC19 and the hydrolysis enzyme APT1(Huang et al. 2023). ML348, an inhibitor of APT1, can restore palmitoylation levels and cure Huntington's disease (HD)(Virlogeux et al. 2021).

## Prenylation

### Prenylation and farnesyl transferase

**Fig. 4:**
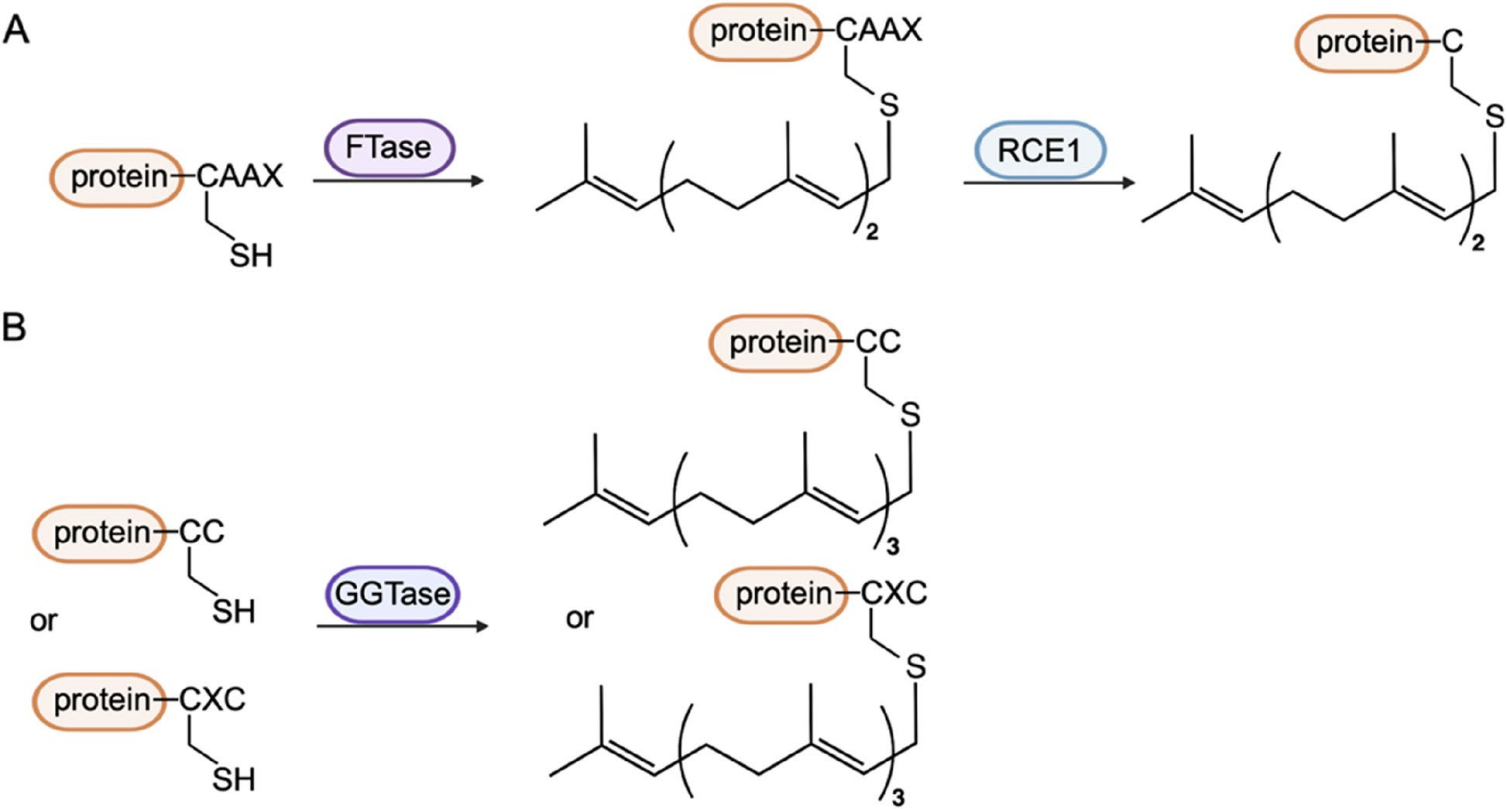
Prenylation process. **A** S-prenylation. **B** S-Geranylgeranylation

Prenylation is an irreversible posttranslational modification that includes S- prenylation and S-geranylgeranylation(Palsuledesai and Distefano 2015; Wang and Casey 2016) (Fig. 4).

S-prenylation is catalysed by farnesyl transferase (FTase) at the C-terminal CAAX box motif (where C represents cysteine, A represents an aliphatic amino acid and X represents any amino acid)(Marchwicka et al. 2022), whereas S-geranylgeranylation is catalysed by geranylgeranyl transferases (GGTase-I,II and III) at a CC or CXC motif(Kallemeijn et al. 2021; Storck et al. 2019). Then, some CAAX proteins undergo further processing by Ras-converting CAAX endopeptidase 1 (RCE-1) to cleave the C-terminal-XXX residues(Zhang and Casey 1996) and isoprenylcysteine carboxylmethyltransferase (ICMT) to facilitate the binding of the carboxy-terminal Cys residue with a methyl group(Wang and Casey 2016). Prenylation has been found in autophagy-related proteins (Table 3).

**Table 3:** Proteins subject to prenylation that regulate autophagy

lipidation	autophagy-related protein	autophagy	disease
prenylation	RAB1(Webster et al. 2016)	unknown	amyotrophic lateral sclerosis and frontotemporal dementia (C9ALS/FTD)
	RAB5(Romagnoli et al. 2018)	unknown	tuberculosis
	RAB7(Guo et al. 2022)	up	lung cancer
	RAB24(Seitz et al. 2019; Ylä-Anttila et al. 2015)	up	Non-alcoholic fatty liver disease (NAFLD)
	RAB32(Haile et al. 2017)	unknown	multiple sclerosis
	RHEB(Sciarretta et al. 2012)	unknown	acute myocardial infarction (MI)
	RALB(Bodemann et al. 2011; Simicek et al. 2013; Dong et al. 2024)	unknown	colorectal cancer
	PTP4A3/PRL-3(Huang et al. 2014)	up	ovarian cancer

### Prenylation of autophagy-related proteins

#### Prenylation of small GTPases

Prenylation is essential for the proper cellular activity of proteins, including Ras family GTPases and heterotrimeric G-proteins(Palsuledesai and Distefano 2015). Specifically, many small GTPases have been reported to be autophagy-related proteins. For example, RAB1, RAB5, RAB24 and RAB32 are essential for the formation of autophagosomes(Hutagalung and Novick 2011), whereas RAB7 is pivotal for the maturation of autophagosomes. These proteins are all prenylated proteins(Khosravi-Far et al. 1991; Wesenbeeck et al. 2007; Alexandrov et al. 1994).

RAB7 is a late endosome protein that is responsible for autophagosome maturation. Autophagosome–lysosome fusion can be impaired by the inhibition of RAB7 prenylation, which leads to the defective localization and function of RAB7(Ranieri et al. 2018).

RAB24 is highly upregulated in the livers of obese patients with nonalcoholic fatty liver disease (NAFLD). RAB24 is related to autophagy, and the prenylation of Rab24 is necessary for the localization of Rab24 to autophagic vacuoles(Ylä-Anttila et al. 2015).

RHEB is a GTP-binding protein that negatively regulates cardiomyocyte survival during nutrient starvation and prolongs ischaemia through mTORC1 activation and autophagy inhibition(Sciarretta et al. 2012). Deubiquitylated RALB(Simicek et al. 2013) promotes the assembly of the RALB-EXO84-beclin-1 complex, which drives autophagosome formation(Bodemann et al. 2011). Butyrate facilitates autophagy via the OR51E1/RALB axis to induce colorectal cancer (CRC) cell death(Dong et al. 2024). RHOA and RAC1 have regulatory functions in starvation-mediated autophagy, but RHOA plays a promotive role, whereas RAC1 plays a suppressive role(Aguilera et al. 2012). Simvastatin treatment leads to increased levels of the unprenylated Ras homologue gene family, member A (RHOA), Ras-related C3 botulinum toxin substrate 1 (RAC1) and cell division cycle 42 (CDC42), which then leads to the activation of the downstream signalling cascade involving superoxide production, JNK activation and Bim-EL upregulation, demonstrating the tremendous potential of simvastatin to induce cancer cell death(Zhu et al. 2013). However, reports describing the direct role of the prenylation of these proteins in autophagy are rare.

#### Prenylation of PTP4A3/PRL-3 is vital for the conversion of LC3-I into LC3-II and increases autophagic flux

PTP4A3/PRL-3 promotes a variety of oncogenic processes, including cell proliferation, invasion and cancer metastasis. Huang et al. demonstrated that PTP4A3/PRL-3, which is a plasma membrane and endosomal phosphatase, requires both catalytic activity and prenylation to convert LC3-I into LC3-II and increase autophagic flux(Huang et al. 2014). Prenylation of PTP4A3 at Cys170 promotes AP accumulation via the canonical PIK3C3-BECN1 autophagy pathway, which is beneficial for the proliferation of ovarian cancer cells (Huang et al. 2014).

#### Inhibition of Icmt induces autophagy-dependent apoptosis

Icmt (isoprenylcysteine carboxylmethyltransferase) is an enzyme that catalyses the final step of the C-terminal processing of isopreneylated proteins. Inhibition of Icmt causes cell death through autophagy-dependent apoptosis, which impairs tumour growth(Wang et al. 2010).

## Glycosylphosphatidylinositol (GPI) anchor

The glycosylphosphatidylinositol (GPI) anchor is a complex glycolipid highly conserved in eukaryotes and is composed of an amino group of EtNP at the end of GPI that is attached to the C-terminus of the protein via an amide bond(Kinoshita 2020; Saha et al. 2016). The GPI anchor can regulate the transport, localization and function of the proteins. For example, Cripto-1 (CR-1)/Teratocarcinoma-derived growth factor 1 (TDGF-1) is a cell surface glycosylphosphatidylinositol (GPI)-linked glycoprotein that functions as an anchor to lipid rafts and endosomes(Klauzinska et al. 2014). Moreover, a lack of the GPI anchoring in PGAP3 causes disorders such as hyperphosphatasia, which is also referred to as Mabry syndrome(Howard et al. 2014).

Nevertheless, the direct function of GPI in autophagy has been rarely reported.

## Cholesterylation

Cholesterylation is a type of autocatalytic lipidation in which cholesterol is attached to proteins via an ester linkage. Cholesterylation was once considered a symbol of hedgehog proteins (Hh)(Kallemeijn et al. 2021; Ciepla et al. 2014; Hentschel et al. 2016), and the cholesterylation of Hh is crucial in development and tumorigenesis(Jiang and Hui 2008). However, only the cholesterylation of smoothened (SMO)has been identified thus far(Xiao et al. 2017), which is a therapeutic target of Hh-pathway-related cancers(Hu et al. 2022). Yao et al. demonstrated that Hh signalling activates AMPK via SMO to promote autophagy and lipid degradation in hepatocytes(Yao et al. 2023), but they did not mention cholesterol modification. To date, the relationship between cholesterol modification and autophagy has not been described.

## Chemical and biochemical methods for identifying the lipidation of proteins

**Fig. 5:**
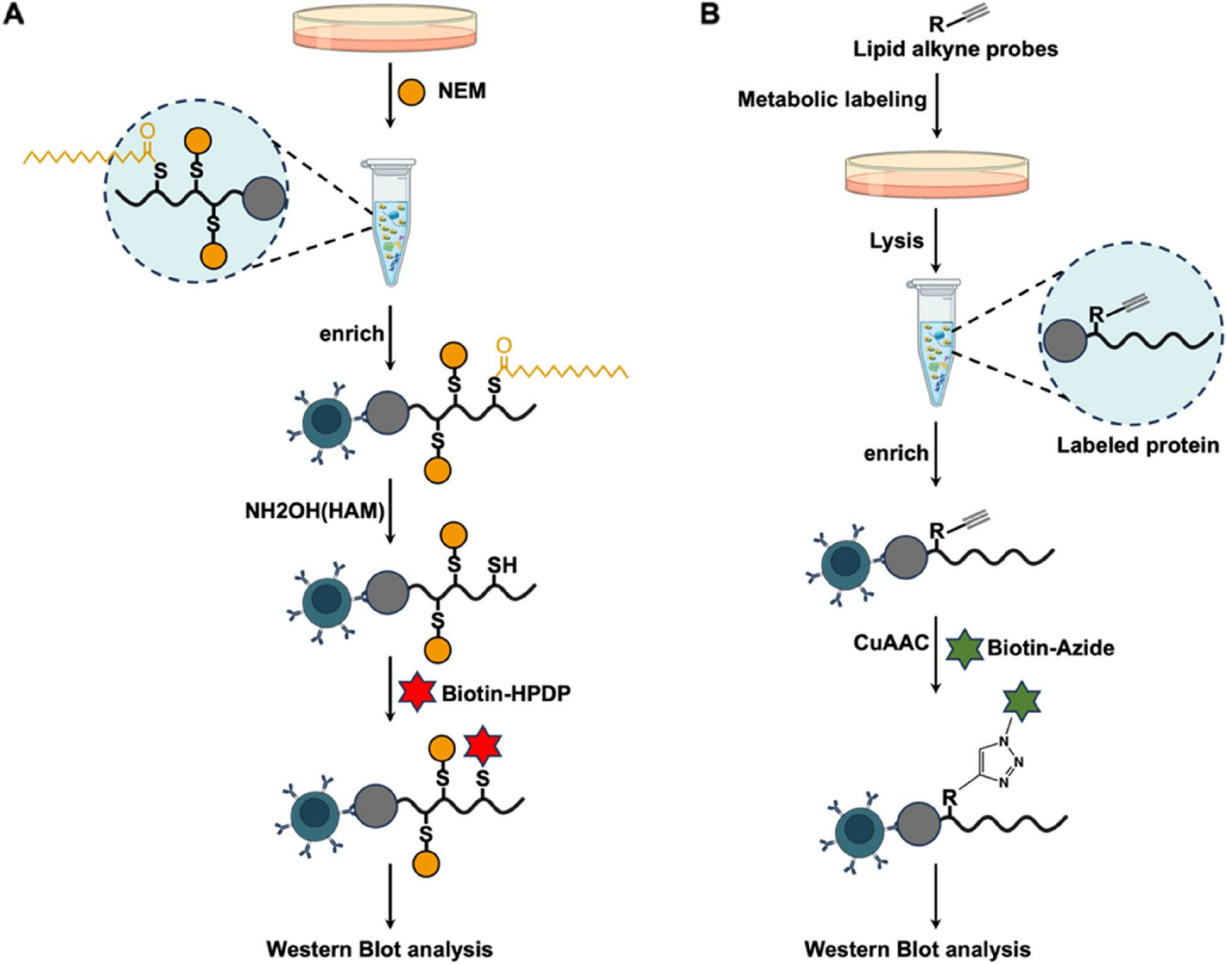
ABE (**A**) and click chemistry (**B**) methods

In the past, radioactive isotope-labelled lipids were widely used to analyse protein lipidation. For example, the incorporation of radioisotope-labelled palmitic acid is still the gold standard for identifying S-palmitoylation(Schmidt and Schlesinger 1979; Schlesinger et al. 1980; Swarthout et al. 2005; Schmidt et al. 1979). However, this method is gradually being phased out because of the use of hazardous reagents, high cost, low sensitivity, lengthy detection periods and environmental impact. Therefore, other more effective and safe methods have been developed, including acyl-biotin exchange (ABE) and click chemistry (Fig. 5).

Acyl-biotin exchange (ABE), which converts the acyl modification to a stable biotin adduct for detection, is one of the most well-known methods for detecting the S-acylation of cysteines. In this method, free thiols on the cysteine residues of proteins are first blocked with N-ethylmaleimide (NEM). Next, the target proteins are purified and enriched. NH_2_OH is subsequently used to remove the thioester bonds of S-palmitoylated cysteine residues. After that, the newly exposed thiols are captured by the biotinylated probe biotin-N-[6-(biotin amido)hexyl]-3′-(2′-pyridyldithio) propionamide (biotin-HPDP). Finally, lipidation is detected by WB or LC‒MS(Forrester et al. 2011; Percher et al. 2016). However, the high false-positive rate and inaccurate localization of the modification site in this method greatly limit its use in research on palmitoylation-modified proteins. Moreover, this method lacks universal applicability across all the types of lipidation.

The click chemistry reaction involves biorthogonal chemical probes and a highly efficient copper(I)-catalysed cycloaddition reaction(Wang et al. 2003). Through click chemistry, a new method has emerged for the identification of protein lipidation via the use of different biorthogonal chemical probes, including terminal alkyne or azido (ω-alkyne or ω-azido) lipid derivatives (fatty acids, sterols, and isoprenoids)(Hannoush and Sun 2010). First, a specific chemical probe is metabolically attached to the protein. Subsequently, antibody-conjugated beads are employed to enrich the target proteins. Then, the proteins tagged with chemical probes can be captured by biotin azide through the click reaction. Finally, the biotinylated proteins are detected via WB or LC‒MS(Hannoush and Arenas-Ramirez 2009). The method of click chemistry-based metabolic labelling with different probes has been widely applied to the global analysis of N-myristoylation(Broncel et al. 2015), S-palmitoylation(Huang et al. 2023), S-prenylation(Hannoush and Sun 2010), cholesterylation(Ciepla et al. 2014), and GPI-anchors(Kundu et al. 2023; Kapoor et al. 2018; Lu et al. 2015) in proteins.

## Conclusions

Lipidation substantially increases the hydrophobicity of autophagy-related proteins, influencing their conformation, stability, membrane association and binding affinity. Many studies have revealed that deregulation of the lipidation of autophagy-related proteins is linked to autophagy dysfunction and various diseases, especially neurodegenerative diseases, cancer and inflammatory disorders((Kenific and Debnath 2015; Sun et al. 2022; Martin et al. 2014; Malek et al. 2012)). For example, the myristoylation of LAMTOR1 at Gly2 increases the stability of LAMTOR1 and its lysosomal localization, which is critical for mTORC1 activation, autophagy and bladder cancer growth (Sun et al. 2022). The palmitoylation of ARMC3 at C507 C518 is important for its ability to promote autophagic activity during spermiogenesis and facilitate the autophagic degradation of ribosomes, which are essential for sperm motility and fertility(Lei et al. 2021). Prenylation of PTP4A3 at Cys170 promotes AP accumulation via the canonical PIK3C3-BECN1 autophagy pathway, which promotes ovarian cancer cell proliferation(Huang et al. 2014). A lack of a GPI anchor on PGAP3 causes disorders such as hypophosphatasia(Howard et al. 2014). The cholesterylation of Hh is crucial in development and tumorigenesis(Jiang and Hui 2008). However, the specific molecular links between GPI and autophagy and between cholesterol and autophagy remain unknown.

The relationship between lipidation and autophagy has now been clearly described in eukaryotes from yeast to humans, suggesting that the lipidation of autophagy-related proteins is a promising target for the treatment of autophagy-associated diseases. However, many challenges remain to be overcome by researchers.

## Data Availability

Not applicable.
